# Recent Applications of Coaxial and Emulsion Electrospinning Methods in the Field of Tissue Engineering

**DOI:** 10.1089/biores.2016.0022

**Published:** 2016-08-01

**Authors:** Phillip McClellan, William J. Landis

**Affiliations:** Department of Polymer Science, The University of Akron, Akron, Ohio.

**Keywords:** coaxial electrospinning, drug delivery, emulsion electrospinning, tissue engineering

## Abstract

Electrospinning has emerged as an effective method of producing nanoscale fibers for use in multiple fields of study. One area of significant interest is nanofiber utilization for tissue engineering because the nanofibrous mats can mimic the native extracellular matrix of biological tissues. A logical next step is the inclusion of certain molecules and compounds to accelerate or increase the efficacy of tissue regeneration. Two methods are under scrutiny for their capability to encapsulate therapeutic compounds within electrospun nanofibers: emulsion and coaxial electrospinning. Both have advantages and disadvantages, which need to be taken into careful consideration when deciding to use them in a specific application. Several examples are provided here to highlight the vast potential of multilayered nanofibers as well as the emergence of new techniques to produce three-dimensional scaffolds of nanofibers for use in the field of tissue engineering.

## Introduction

### History and background information about electrospinning

The technique known as electrospinning was discovered and described over a century ago^1^ as a derivative of electrospraying, a method that utilizes electrostatic forces to generate polymer droplets.^2–5^ At the outset of electrospinning development, at least 11 patents were issued to Formhals over the course of ∼10 years (1934–1944) for various designs providing a method to generate fine polymer filaments.^6–16^ In 1964, Geoffrey Taylor published research which explained the deformation of water droplets in the presence of an electric field.^17^ His use of mathematical treatment and imaging demonstrated the distortion of spherical drops into conical shapes^17^ and, in 1969, he presented another article focusing on the ejection and formation of fine jets from viscous solutions.^18^ Annis et al. spent time in the late 1970s investigating electrospun polyurethane for potential vascular prostheses,^19^ but, overall, electrospinning was not considered to have many useful applications. The technique languished for many years until the early 1990s when interest was renewed largely as a result of the need for a process which would enable the fabrication of fibers on the nanometer scale. In this regard, Reneker, Yarin, and other researchers began to examine more closely the fibers that were formed by this process and investigate the physics governing their formation.^20–31^

**FIG. 1.**
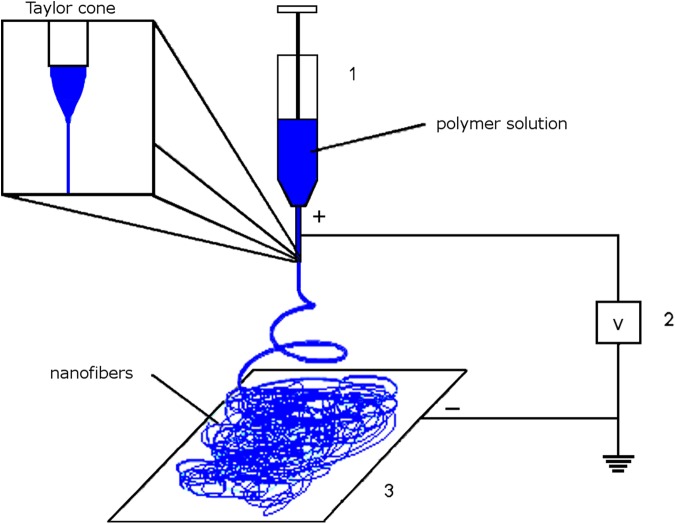
Basic electrospinning apparatus. A polymer solution is loaded into a syringe (1) with a needle attached to a high voltage power supply (2). The nanofibers generated accumulate on the surface of a collector (3) connected to an oppositely charged electrode of the high voltage power supply. A flow-modified Taylor cone is highlighted in the upper left of the figure.

Electrospinning is possible because of an imbalance between two physical properties common to the process, surface tension, and electric field strength. A solution or melt with sufficiently high viscosity passes through an orifice, usually at the end of a needle or metal cone with a small hole at its tip, to generate a small droplet (Fig. 1). When surface tension dominates intermolecular interactions, the droplet of solution at the tip of the needle or cone is stable and remains in place. As the electric field strength increases, excess electrical charges, either positive or negative, accumulate on the surface of the suspended droplet and begin to overcome the surface tension. A Taylor cone^17,18^ is formed as the drop distorts and a small strand of solution, or jet, is ejected from its tip, generating a flow-modified Taylor cone. The excess electrical charges within the jet repel one another, the result of which leads to rapid elongation of the expelled strand of solution. The jet of solution travels to a collector, which is usually in the form of a flat, electrically conductive plate. As the strand of solution travels to the collector, the solvent evaporates to leave solid fibers.

Many parameters may be manipulated to optimize the electrospinning process. A few factors often altered when solvent-based methods are employed include applied voltage, distance between the electrospinning tip and collector, solvent volatility, electrical conductivity of the solution, and solution viscosity. These parameters have significant effects on the morphological characteristics of the formed nanofibers. Environmental factors such as temperature and humidity may also have a marked effect on the morphology of the fibers formed,^20,21^ and they have led to the creation of electrospinning designs that have tips and collectors either submerged in liquid^32^ or sealed in chambers with tightly controlled atmospheric conditions.^33^ Melt-based electrospinning is manipulated by some of the same factors as solvent-based electrospinning (applied voltage, distance between tip and collector, electrical conductivity of the melt), but it can also be modified by altering other aspects, such as the temperature of the melted polymer.

### Potential uses for electrospun nanofibrous mats

A variety of nonwoven nanofiber mats may be generated by electrospinning. These have been utilized in applications for wound dressings,^34–36^ filtration devices,^37^ protective clothing and textiles,^38^ sensors,^39^ tissue scaffolds,^40,41^ and drug delivery systems.^33,42,43^ All of these areas benefit from the high surface area-to-mass ratio of the nanofiber mats. As an example, it is interesting to consider drug delivery in this context. A significant problem encountered in drug delivery is maintaining a therapeutic dosage to a patient over a specified period of time. Controlling the dosage rate is especially important in the case of drugs with a low therapeutic index, such as doxorubicin (dox)^42^ or digoxin,^44^ as they must be administered more often and in smaller amounts to minimize the risk of toxic side effects and remain within the window of concentration where they will be effective. However, as the frequency of required doses increases, patient compliance generally decreases. An effective way in which to ensure patients will adhere to the dosage schedule is to place the therapeutic agent within a device or chemical compound that will control the rate at which the agent is released and decrease the frequency of required doses. Insulin pumps, nicotine patches, and time-release tablets are a few examples of products currently in the consumer marketplace which utilize a form of controlled drug delivery. Hydrogels,^45–47^ dendrimers,^48–51^ liposomes,^52–55^ and microelectromechanical systems (MEMS)^56–59^ represent a small fraction of technologies under investigation for their use in drug delivery.

One of the advantages of utilizing electrospun nonwoven nanofiber mats for drug delivery derives from the capability to employ them as wound dressings or tissue scaffolds for tissue engineering. The therapeutic agent can be loaded into the electrospun fibers and released at a steady rate over a period of time. Early biological applications of electrospun fibers concentrated on the field of surface wound repair for a few reasons. First, the materials used can be nonbiodegradable and consist of polymers which are already designed to form nanofiber mats.^35,36^ Second, the electrospun mats do not need to be subjected to the same rigorous testing protocols as a product used for implantation into the human body. Third, the morphological characteristics of the nanofibrous mats can be adjusted to allow air to pass through the mats while controlling or preventing the flow of moisture or dirt.^38^ Perhaps the most interesting feature of the nanofiber mats is the ability to couple them with antibacterial or regenerative agents to increase the rate of healing.

Small molecules or particles may become trapped within nanofibers as the solutions are electrospun. For instance, titanium dioxide nanoparticles have been added to nanofibers of polyurethane, which demonstrated increased antibacterial activity and significant water vapor permeability, but exhibited relatively low cytotoxicity to cultured L929 (mouse fibroblast) cells. Water vapor permeability was examined because the desired application of these nanofibers was the treatment of burn wounds, which require moisture to prevent dehydration and promote tissue regeneration.^35^

Skin ulcers associated with Leishmaniasis were treated effectively through the use of electrospun mats which were designed to release nitric oxide upon the addition of a small amount of water.^36^ The primary issue encountered in utilizing nonbiodegradable materials is that they remain in place if they are implanted into the human body. To circumvent this problem, biodegradable polyesters such as polyglycolic acid (PGA), poly-l-lactic acid (PLLA), and polycaprolactone (PCL) were examined with the intent of creating biodegradable nanofibers that may be implanted into the human body and dissolved slowly over time with few, if any, deleterious effects.

## Tissue Scaffolds

**FIG. 2.**
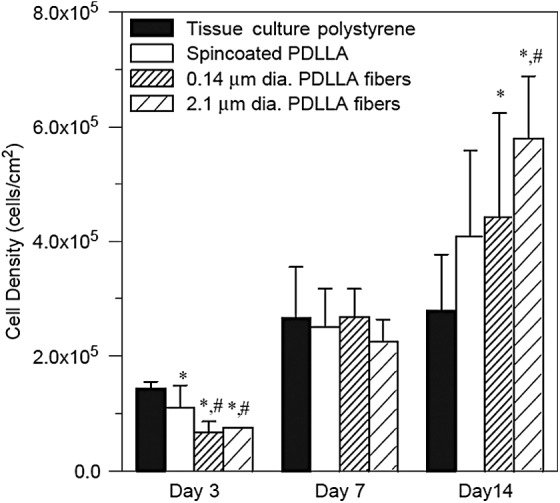
Plot comparing the growth of MC3T3-E1 (mouse calvaria-derived osteoprogenitor) cells in monolayer and on nanofibers. Statistically significant differences from *tissue-culture polystryrene and ^#^spin-coated PDLLA are noted. PDLLA, poly-d,l-lactic acid. (Reprinted with permission from Elsevier from Ref.^69^).

**FIG. 3.**
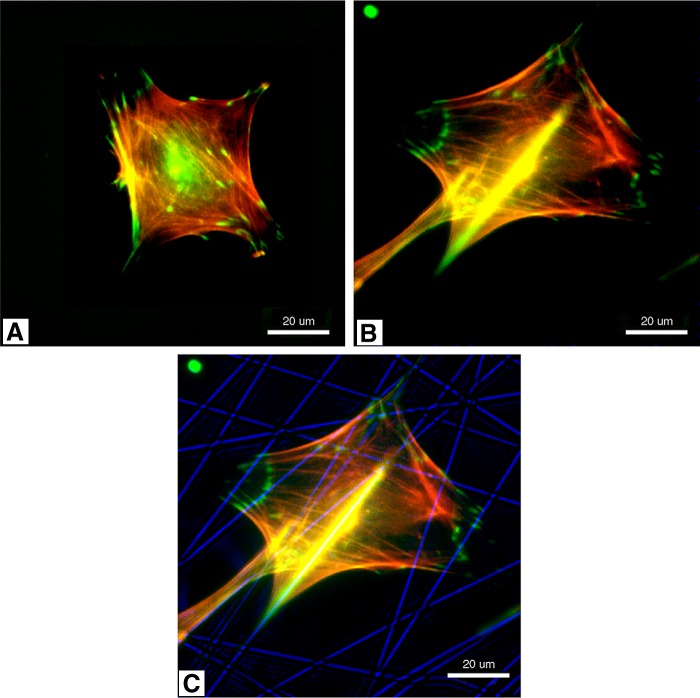
Immunofluorescent staining of adherent cells (MC3T3-E1) on spin-coated PDLLA **(A)** and PDLLA nanofibers **(B)**. Green and red indicate vinculin and actin, respectively. **(C)** Shows the immunofluorescent staining image **(B)** superimposed onto a phase contrast image of PDLLA fibers (blue). (Reprinted with permission from Elsevier from Ref.^69^).

Synthetic, bioresorbable materials, such as polyethylene oxide (PEO),^42^ PGA,^60^ PCL,^43^ polylactic acid (PLA),^42,43,60^ and various copolymers of these compounds have been utilized to great effect to form tissue scaffolds of electrospun nanofibrous mats. Natural polymers, such as collagen and elastin, have also been successfully electrospun to provide nanofiber mats that closely resemble those components of the extracellular matrix of some biological systems.^61^ Chitosan,^62–65^ alginate,^65,66^ and hyaluronan^67,68^ are more natural materials, which have gained interest in light of their availability, affordability, and cellular compatibility. Whether synthetic or natural materials are used for electrospinning, living cells appear to attach and proliferate more readily on the nanofiber mats than when grown in monolayer culture.^69–71^ Figure 2 provides a graphical representation comparing cells grown in monolayer (spincoated poly-d,l-lactic acid [PDLLA]) and those grown on nanofibers (PDLLA fibers). The results for day 3 show a lower number of cells initially attached to the nanofibers. However, by day 14, the cell density on the nanofibrous materials has increased significantly compared with the cell density present on the spin-coated PDLLA. Figure 3 illustrates the difference in cell morphology when cells are grown in the presence of three-dimensional nanofibrous PDLLA. The cell shown in the second and third images is utilizing the nanofibers (shown in blue in the third image) as its external support structure.

Significant advances have been realized in recent years with regard to the utilization of electrospun nanofiber mats composed of biodegradable materials for tissue engineering. The relative simplicity of the electrospinning technique, coupled with the morphological characteristics of the fibrous mats, make it an attractive method for forming tissue scaffolds. By controlling the size, density, composition, and orientation of the fibers, it is possible to produce materials which approximate closely the fibrous structures that are present in the extracellular matrix of almost any tissue.

For tissue engineering, the nanofiber mats containing a therapeutic agent can be utilized as part of a tissue scaffold. In such an application, the electrospun fibers should serve to mimic the three-dimensional environment of the extracellular matrix of the target tissue as well as release small amounts of the desired agent in a controlled fashion. The composition of the fibers may be altered to optimize their drug-loading efficiency, activity, and biocompatibility.

### Aligned PLLA nanofibers for neural tissue engineering

With respect to a specific example, PLLA was selected as a suitable material for fabricating mats of random and aligned nanofibers in at least one case of neural tissue engineering.^72^ The viability of mouse neuronal stem cells was not significantly affected by the alignment of the fibers. However, the alignment of the fibers served to influence the cells to grow in a more directed fashion similar to those found within native nerve tissue. Also, the nerve cells on aligned fibers demonstrated a significant increase in neurite length.

### Collagen and elastin nanofibers for smooth muscle tissue

Attempts to utilize natural polymers, such as collagen and elastin, have been met with success in some instances. The use of collagen/elastin mats for smooth muscle tissue engineering demonstrates promise because the fibers exhibit similar mechanical properties as the smooth muscle tissue located within blood vessel walls.^61^ Vascular grafts could be tailored to specific situations with only minor modifications to the nanofiber collector. Electrospun collagen/elastin mats could also be utilized to repair the outer walls of the stomach or intestinal tract.

### Nanofibrous mats containing calcium carbonate or hydroxyapatite nanoparticles

Wutticharoenmongkol et al. generated nanofibrous mats of PCL that contained calcium carbonate or hydroxyapatite nanoparticles for bone tissue regeneration.^73^ The nanofibrous mats produced were referred to as a “guided bone regeneration membrane” and it was postulated that the nanoparticles would facilitate the proliferation and differentiation of osteoblasts.^73^ Early results indicated that the scaffolds did not exhibit any cytotoxic effects on human osteoblasts or rat fibroblasts.^73^ In addition, the osteoblasts grown on nanofibers containing calcium carbonate were examined under scanning electron microscopy and demonstrated the initial signs of mineralization with the appearance of small crystalline granules. A notable potential consequence of inducing and maintaining osteoblast differentiation was mentioned in the publication. The study found that the osteoblasts which had differentiated slowed considerably with respect to proliferation.^73^ A similar situation was mentioned by Owen et al. in a study of rat osteoblasts grown *in vitro*.^74^

## Core–Sheath Nanofiber Structures

### Emulsion electrospinning

Although the electrospinning technique is relatively simple, difficulties may occur with the method in aspects of loading therapeutic agents into the fibers. Problems may be encountered, for example, with proteins because of their size and requirements relative to their three-dimensional structure. In this regard, small conformational changes could occur as a result of the electrospinning process (solvent exposure, shear stresses) and they may render a protein inactive or possibly even toxic. To circumvent such a situation, proteins may require the use of more complex techniques, such as emulsion or coaxial electrospinning, to be loaded efficiently and effectively into nanofibers to be electrospun. In the case of both emulsion and coaxial electrospinning, the nanofibers generated consist of an outer sheath and inner core of differing composition. Emulsion electrospinning relies on chemical means of separation through the creation of an emulsion within a single solution and the subsequent organization of the emulsified droplets into two distinct phases as the solvent evaporates from the electrospun fibers.^75^ This technique has proven successful in encapsulating nerve growth factor (NGF),^60^ proteinase K,^76^ lysozyme,^77^ cytochrome C,^78^ and bovine serum albumin.^79^ Emulsion electrospinning is also being investigated because it accommodates the use of water as the solvent instead of more hazardous chemicals.^80^ In many cases, surfactants or other amphiphilic compounds are used to generate the emulsified droplets in water-based systems.^60,79,80^

#### Nanofibers of silk fibroin and PCL

Before work with encapsulating therapeutic agents, suitable solution and electrospinning parameters should be determined. For example, core–sheath-structured nanofibers of silk fibroin (SF) and PCL have been constructed utilizing emulsion electrospinning.^81^ SF is similar to collagen in that it is a biopolymer obtained from natural sources and contains cell recognition peptides such as RGD (arginine-glycine-aspartic acid), which facilitate cell attachment and proliferation.^82,83^ The main focus in generating SF/PCL fibers was to capitalize on the mechanical strength of the PCL while exploiting the advantages of SF as a tissue engineering material. Testing *in vitro* using primary fibroblast cells from human skin (FEK4) demonstrated an increase in initial cell attachment as well as a slight increase in cell proliferation rate.

After positive results were obtained from the SF/PCL combination, properties of the nanofibers were optimized to a greater degree by the addition of hyaluronan to the electrospinning solution.^84^ Hyaluronan, or hyaluronic acid (HA), is a glycosaminoglycan found in the extracellular matrix of many tissues and it can promote cellular adhesion and proliferation.^67^ The purpose of its inclusion into the SF/PCL emulsion was to provide some measure of control over the adsorption of proteins to the surface of the nanofibers while still enhancing infiltration of cells into the scaffold. Water swelling and contact angle measurements indicated an increase in hydrophilicity of the nanofibrous scaffolds as a result of HA addition. Also, the rate of FEK4 cell infiltration and proliferation was significantly higher after 5 days when compared with scaffolds composed only of SF and PCL.^84^

One potential challenge for researchers intending to capitalize on the success of this study and others like it results from the use of a fluorinated solvent, in this case, hexafluoroisopropanol (HFIP). HFIP is a corrosive, volatile solvent, which may not be suitable for biological molecules or desired therapeutic agents.^85^ Other solvent systems were developed to combat this issue and can be used to great effect with the emulsion electrospinning methodology.^85,86^

#### Encapsulation and controlled release of doxorubicin

As outlined in the *Emulsion Electrospinning* section, the emulsion electrospinning technique requires a less complex apparatus than comparable coaxial arrangements to generate core–sheath-structured nanofibers. Only one syringe pump and electrospinning tip are required. The core and sheath of the nanofibers can be simply the result of the separation between organic and aqueous phases during the evaporation of the solvent as the fibers are formed. In some cases, an emulsifier such as sodium dodecyl sulfate (SDS) is required to produce a stable water-in-oil or oil-in-water emulsion for electrospinning.^42^ The thickness of the inner and outer layers of the nanofibers can be altered by changing solution parameters as well. When Xu et al. encapsulated dox within PLA/PEO nanofibers, they discovered that increasing the concentration of dox within the core of the fibers shrinks the diameter of the core as a result of the favorable association between dox molecules.^42^ The association and smaller diameter core also had the effect of significantly decreasing the release rate of the drug from 60% to only 20% released within the first 5 h. More importantly, studies such as this one demonstrate the feasibility of producing nanofibers containing aqueous and organic phases. This dual-phase nature is critical not only in the case of small molecule therapeutics, but even more so for proteins and growth factors.

#### Altering cytochrome C release characteristics in PLLA nanofibers

A study by Maretschek et al. demonstrated the encapsulation of a relatively small heme protein, cytochrome C, within nanofibers of PLLA.^78^ The work showed that a change of 1–3% PLLA concentration significantly slowed the initial release of cytochrome C from the core of the fibers, essentially eliminating the burst-release phenomenon.^78^ This investigation highlights the concept that manipulating the release rate of the therapeutic agent can be achieved through altering the sheath of the nanofibers. The medium into which the nanofiber mats are placed can exhibit a marked effect on release characteristics as well. This research group demonstrated that the release rate of cytochrome C could also be altered through the addition of hydrophilic polymers such as polyethyleneimine (PEI) or poly-l-lysine (PLL).^78^ By addition of one of these polymers to the emulsified electrospinning solution, the hydrophobicity of the nanofiber mats was decreased and the release rate of the protein was increased, results which also reintroduced the initial burst-release phenomenon. The capability to manipulate and control the release process shown by this study is critical in many cases for effective administration of therapeutic agents. Such control is especially important with respect to growth factor-centered treatments as it may be beneficial to release certain factors in high concentrations initially and slowly decrease the dosage rate over time.

#### Controlling the release profile of basic fibroblast growth factor in PELA nanofibers

**FIG. 4.**
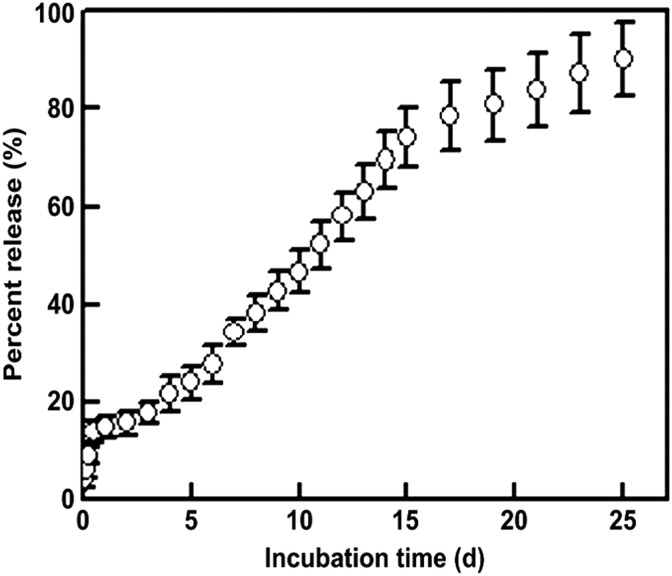
Release characteristics over 25 days *in vitro* for PELA nanofibers loaded with bFGF. Nanofibrous mats of PELA nanofiber containing bFGF (*n* = 3) were suspended in PBS (pH 7.4) in tubes that were placed in a shaking water bath (37°C, 100 cycles/min). PBS was collected and analyzed by ELISA at specified intervals to determine the percent of total bFGF released. bFGF, basic fibroblast growth factor; ELISA, enzyme-linked immunosorbent assay; PBS, phosphate-buffered saline; PELA, polyethylene glycol/poly-dl-lactide. (Reprinted with permission from Elsevier from Ref.^34^).

A more thorough examination of the release profiles for encapsulated proteins was undertaken by Yang et al.^34^ Specifically, this group focused on the release of basic fibroblast growth factor (bFGF) sequestered within polyethylene glycol/poly-DL-lactide (PELA) nanofibers. Figure 4 shows release characteristics of bFGF over a period of 25 days *in vitro*. The initial 12 h after incubation (1 × phosphate-buffered saline, 37°C) of the drug-loaded nanofiber mats demonstrated the burst-release phenomenon, freeing ∼14% of the total bFGF from the nanofibers. Over the next 15 days, another 60% of the growth factor was liberated from the nanofibers during the “fast” phase of release. Following another 10 days, the release rate tapered off significantly and resulted in 15% of the protein being freed from the fibers during the “slow” release phase.

The bFGF-loaded nanofiber mats were tested *in vitro* for compatibility with living cells using mouse embryo fibroblasts. For testing *in vivo*, the growth factor-loaded nanofiber mats were applied to dorsal wounds in diabetic rats.^34^ After 1 week, the wounds covered with the bFGF-loaded PELA nanofibers had already begun to heal, whereas the PELA nanofiber mats without the growth factor and containing free growth factor had not. Within 3 weeks, the wounds covered with growth factor-loaded nanofibers had completely healed. The other testing groups required an additional week to heal, and the control group still had not healed fully at the end of 4 weeks. Such marked success in wound healing was attributed by Yang et al. to three factors: encapsulation of bFGF prevented its early degradation in the proteolytic environment of the wound, the nanofibers kept the factor localized to the wound, and sustained release of bFGF was maintained over time.^34^ In this case, the triphasic release profile of bFGF may have been beneficial to wound healing. The initial burst of the growth factor may facilitate rapid proliferation of fibroblasts to start the healing process. The second and third phases of release follow with decreasing release rates, prolonging the presence of the growth factor within the wound while slowing the proliferation of cells as the wound shrinks and eventually closes.

### Coaxial electrospinning

Coaxial electrospinning differs significantly from emulsion-centered methods in that it generates core–sheath fibers by physical separation through the utilization of two electrospinning tips and two solutions.^72^ Certain processing parameters and solution properties such as inner and outer solution flow rates, viscosities, and electrical conductivities are often taken into account when attempting to apply the coaxial technique. The coaxial electrospinning process can be utilized to create drug delivery systems of varying complexity. For instance, the core and sheath compositions of the fibers could be chosen for strength and cell attachment properties, respectively. The approach has been demonstrated by electrospinning nanofibers with a thermoplastic polyurethane core and a collagen sheath.^87^ Fibers such as these provide desirable mechanical strength as a result of the polyurethane core and facilitate increased cell attachment and proliferation because of the presence of the collagen sheath.

#### Bilayer nanofibers with a polyurethane core and a collagen sheath

**FIG. 5.**
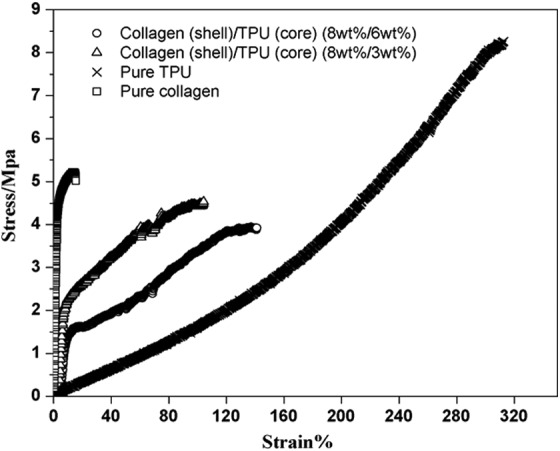
Stress–strain curve for electrospun TPU, collagen, and TPU/collagen coaxial nanofibers highlighting the mixture of physical properties that results from the combination of collagen and TPU in the nanofibers. TPU, thermoplastic polyurethane. (Reprinted with permission from Elsevier from Ref.^87^).

As noted above, one of the main advantages of fabricating a tissue scaffold of nanofibers with a core–sheath structure is the capability to combine materials of varying mechanical and chemical properties. An example of such a combination was demonstrated by Chen et al., who produced nanofibers, which were composed of a polyurethane core and a collagen sheath.^87^ The polyurethane core contributed significant mechanical strength to the nanofiber mats, which would not have been realized using a pure collagen scaffold. Stress–strain curves (Fig. 5) generated by tensile test measurements showed that the collagen-coated polyurethane fibers were not as strong as pure urethane fibers, but they were significantly more robust than nanofibers of pure collagen. The decrease in mechanical strength was acceptable because a scaffold composed purely of synthetic polyurethane would not exhibit the same cell recognition and attachment as one fabricated with collagen. Studies *in vitro* utilizing pig iliac endothelial cells demonstrated a marked increase in the number of viable cells adherent to the scaffolds after 1, 3, and 5 days of cell culture when compared with polyurethane nanofibers alone, polyurethane fibers bulk-coated with collagen, and glass cover-slips.^87^ It is important to note that any nanofibers fabricated with collagen on their surface typically require an extra step to crosslink the collagen and preserve the structure of the nanofibers.^61,87,88^ Overlooking this critical step will result in dissolution of the collagen into aqueous medium.

#### Production of poly(glycerol sebacate) nanofibers

The coaxial technique has been employed to produce single-phase, solid nanofibers from materials which would be extremely troublesome to electrospin under normal conditions.^89^ Poly(glycerol sebacate) (PGS) is a degradable synthetic material which exhibits mechanical properties similar to elastin.^90^ Nanofibers of PGS are difficult to produce under basic electrospinning conditions because they require curing after the fibers are produced to generate the crosslinks which allow the material to display three-dimensional morphologies similar to elastin and collagen. During the curing process, the nanofibers need to remain separated from one another to prevent them from melting and merging into larger fibers or thin films. Yi and LaVan were successful in generating nanofiber mats of PGS by utilizing PLLA as a sheath to sequester the uncured PGS and ensure it would crosslink while remaining as nanofibers.^90^ Once the PGS was crosslinked, the PLLA was removed using dichloromethane to leave a nanofibrous mat of pure PGS. They proposed using the PGS nanofiber scaffolds for tissue engineering in microvasculature and showed the nanofibers had no significant effects on viability of human dermal microvascular endothelial cells.^90^

#### Encapsulation of NGF in PLGA nanofibers

**FIG. 6.**
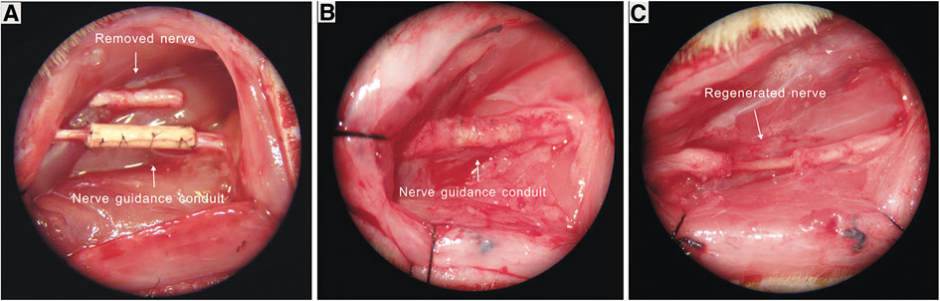
Surgical implantation of an aligned NGC for nerve regeneration in a rat model of a 13-mm sciatic nerve defect viewed under a microscope. **(A)** The NGC was used to bridge the 13-mm nerve defect; **(B)** the appearance of the NGC 12 weeks after implantation; **(C)** the regenerated nerve after removing the NGC. NGC, nerve guidance conduit. (Reprinted with permission from Taylor and Francis from Ref.^90^).

Research groups have demonstrated methods for encapsulating successfully NGF in poly(lactic-co-glycolic acid) (PLGA)^91^ and vascular endothelial growth factor (VEGF) within Dextran/PLGA^92^ as well. In both cases, the encapsulated growth factors maintained significant bioactivity and were not altered by the coaxial electrospinning process. In the instance of NGF encapsulated within PLGA, Wang et al. demonstrated a controlled, linear-release profile following an initial burst-release of 29.5%.^91^ Also, the nanofibrous mats were electrospun in an aligned fashion and rolled into small tubes for testing as a potential nerve guidance conduit (NGC). Results from studies *in vivo* indicated the NGF-loaded nanofibers were as effective as autograft nerve tissue for regenerating tissue in a rat sciatic nerve model.^91^ Figure 6A shows the insertion of the NGF/PLGA tube in a rat having a 13-mm defect introduced into its sciatic nerve. In Figure 6B, the NGC is shown 12 weeks after the initial implantation. The conduit is completely encapsulated by this time. The regenerated nerve is illustrated in Figure 6C, where the NGF/PLGA tube has been removed from the sciatic nerve of the rat at the 12-week time point. In addition to demonstrating the effectiveness of encapsulation of NGF within nanofibers, this study also highlighted short-term effects *in vivo*. No significant adverse health effects were noted throughout the course of the experiment. It appeared that the NGF was released locally around the nerve defect and did not have a systemic effect.^91^

#### Nanofibers of dextran/PLGA loaded with VEGF

**FIG. 7.**
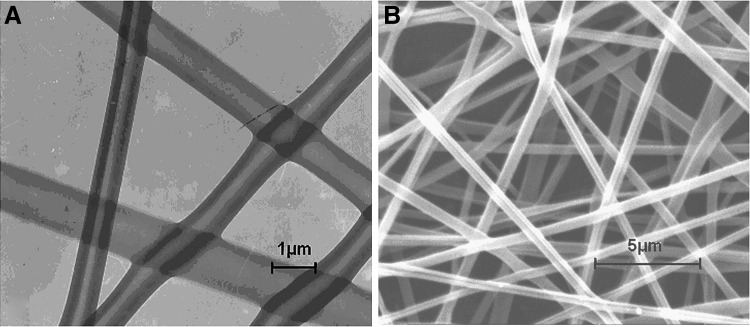
Photomicrographs from transmission **(A)** and scanning **(B)** electron microscopy of DEX(VEGF)/PLGA fibers prepared by coaxial electrospinning. DEX, dextran; PLGA, poly(lactic-co-glycolic acid); VEGF, vascular endothelial growth factor. (Reprinted with permission from Taylor and Francis from Ref.^92^).

In a separate study, dextran/PLGA nanofiber mats loaded with VEGF were tested for cell proliferation and growth factor viability using human umbilical vein endothelial cells.^92^ It was noted that the cells began to infiltrate the pores of the VEGF-loaded nanofibrous mats as soon as day 3 after seeding and commenced secreting extracellular matrix, which began to be incorporated with the nanofibers to provide a suitable environment for additional cell growth.^92^ Figure 7 shows transmission and scanning electron micrographs (TEM and SEM, respectively) of coaxially spun nanofibers. The TEM micrograph (Fig. 7A) emphasizes the “hollow” or tube-like nature of the spun fibers, whereas the SEM image (Fig. 7B) demonstrates the uniformity of the nanofiber size distribution. Both of these factors are critical for controlling drug-loading and release characteristics using nanofibers. Nanofibers having nonuniform diameters and a high degree of variability with respect to the thickness of the walls of the tube could exhibit unpredictable drug-release profiles.

Another significant advantage of coaxial electrospinning for tissue scaffolds and controlled drug delivery is seen in an experiment conducted using myoblasts isolated from the skeletal muscle of mice.^93^ Coaxial nanofibers with a core containing VEGF and a shell composed of polyurethane were produced and demonstrated more efficient and even loading of the factor into the nanofibers when compared to simple electrospinning. Also, sustained release of the factor occurred over the course of 30 days with initial burst release occurring over the first 6 days in culture. Cell-seeded constructs were implanted into hemophilic mice. The skeletal muscle-engineered constructs demonstrated marked improvement compared with the control groups. Histological examination showed an increase in vascularity and presence of significantly more and larger lymphatic vessels.^93^

## Comparing Emulsion and Coaxial Electrospinning Techniques and Potential Applications

Emulsion, compared to coaxial, electrospinning is the more prevalent technique because it provides protein solvation within a mild solvent and separation from the harsh solvent required to dissolve the desired polymer.^43,75,77^ Emulsion electrospinning also has the advantage of simplicity, but it lacks well defined control over the placement of the therapeutic agent within either the core or shell of the structure. Enabling precise control over the location of the drug within the core or shell of the nanofibers is one of the advantages of using coaxial electrospinning instead of emulsion.^94^ Reproducibility and high-throughput are two other advantages in designing drug delivery devices using coaxial electrospinning. The major disadvantage with coaxial electrospinning lies in the complexity of its setup. A coaxial needle and multiple syringe pumps are required to control the precise formation of the core–sheath structures. The syringe pumps are relatively simple to arrange for effective delivery of polymer solutions. The design of the coaxial needle is critical to the ultimate arrangement of the core–sheath fibers. The simplest and most often utilized needle arrangement is a single core centered within the outer sheath.^69,87^ More complex strategies place multiple needles within the center to produce nanofibers having more than a single core.^95–97^ The fabrication of intricate needle designs such as these serves to add costs to the initial setup of the coaxial electrospinning apparatus and is investigated in situations where a simpler technique is not viable.

Utilization of the coaxial electrospinning technique could allow creation of more complex drug delivery systems as well.^97^ For example, the inner layer of the electrospun fibers could be composed of PCL, loaded with osteogenic protein-1 (OP-1), whereas the outer layer could consist of collagen, loaded with bFGF. The reason for utilizing OP-1 and bFGF in this case is that OP-1 has been shown to promote differentiation of chondroblasts and osteoblasts^98^ and bFGF has a demonstrated capability to increase cell proliferation.^99,100^ Conceptually then, bFGF loaded into the outer sheath of the nanofibers would be released into the tissue environment before OP-1 encapsulated within the core of the fibers. Thus, this type of scaffold could promote delivery of factors or drugs in two stages, the first for enhancing cell proliferation and the second for ensuring that the cells differentiate into the desired cell type. A potential pitfall, which could hinder this approach, might result from the use of PCL as the material for the inner layer and the application of an organic solvent required for PCL dissolution. The organic solvent selected could alter the activity of OP-1, potentially reducing or eliminating the advantageous effects of its incorporation into the nanofibers.

Release profiles of OP-1 and bFGF should be governed by the thickness of the inner core and outer sheath as well as the chemical composition of each electrospun fiber layer.^77,78^ As the nanofiber degradation will not be completely uniform, an overlap in the release profiles between OP-1 and bFGF may be present. It should be possible to adjust this window of overlap to maintain an optimal ratio of the two factors. Such an approach would be beneficial in many applications which require multiple drugs to be released in a sequential manner.

**FIG. 8.**
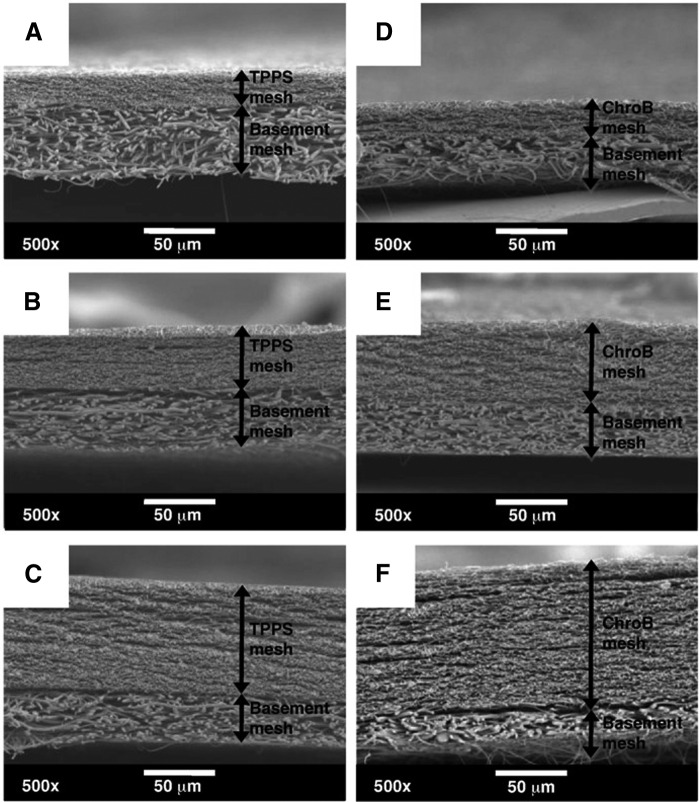
Scanning electron micrographs of layered mesh scaffolds composed of PLCL and loaded with multiple therapeutic agents. TPPS-loaded and ChroB-loaded meshes with different mesh thickness. **(A–C)** TPPS meshes with 23 ± 2 μm, 40 ± 3 μm, and 81 ± 4 μm thickness, respectively. **(D–F)** ChroB meshes with 28 ± 5 μm, 58 ± 3 μm, and 103 ± 5 μm thickness, respectively. PLCL, poly-l-lactide-co-ɛ-caprolactone; TPPS, 5,10,15,20-tetraphenyl-21H,23H-porphinetetrasulfonic acid, disulfuric acid; ChroB, chromazurol B (2,6-dichloro-4′-hydroxy-3′,3″-dimethylfuchsone-5′,5″-dicarboxylic acid disodium salt). (Reprinted with permission from Elsevier from Ref.^101^).

Research has already demonstrated the capability to fabricate multidrug nanofiber mats using sequential electrospinning to generate a multilayered mesh (Fig. 8).^101^ The approach required two extra layers of fibers to delay the release of the second drug and produced fibers of differing morphologies in all four layers. As the fibers in every layer were composed of poly-l-lactide-co-ɛ-caprolactone (PLCL), macroscopic separation of the mesh layers did not appear to be an issue. However, the multilayered mats were being examined principally for applications in sequential chemotherapy, not in tissue engineering.^101^ Therefore, the meshes were not created to optimize attachment and proliferation of cells. Also, using multilayered fibers could prove to be problematic in tissue engineering applications because the cells may infiltrate all the layers and begin degrading them simultaneously, resulting in premature release of the second drug. This situation could be prevented, in concept, by generating nanofibrous mats having sufficient fiber density and small pore size to prevent rapid infiltration of cells. Small pore electrospun mats that hinder cell infiltration might be advantageous in wound healing applications, for example, in which growth factors and related molecules could penetrate a fiber mesh relatively easily, whereas cells, bacteria, and other such larger elements could not. Considerations of fiber size and porosity are important related to diffusion of various molecules and infiltration of cells into electrospun mats for tissue engineering applications. Discussion of these factors has been presented in multiple studies.^69,102–105^

In any case, the aforementioned applications of coaxial electrospinning in tissue engineering and drug delivery increase the complexity of the basic technique. Controlling the release profiles and thickness of the inner and outer electrospun fiber layers may be challenging, for instance. Any therapeutic agents loaded into the electrospinning solutions must also remain active after being subjected to the various organic solvents and the electric field in the electrospinning operation. As a beneficial side effect, the sequestration of a drug within electrospun nanofibers could prolong the amount of time a drug could be stored.^106^ This possibility is of particular interest for many therapeutic formulations in the pharmaceutical industry. Long-term factor or drug storage can be a significant obstacle to production of compounds, which are not required on a regular basis or require transport to locations lacking the necessary infrastructure to preserve them. Proper preparation is still important when storing or transporting therapeutic agents in this fashion. For instance, PCL, PLA, and PGA undergo hydrolytic degradation under normal environmental conditions. It would, therefore, be prudent to store drug-loaded nanofibers containing such compounds in an environment with very low humidity. While significant advances are being made in developing water-soluble^107,108^ and chemically stable solutions,^109–111^ the pharmaceutical industry will benefit greatly from the introduction of simple preservation methods to prolong the useful life and bioavailability of unstable, yet effective, formulations.

### Current challenges in designing drug delivery systems utilizing electrospun nanofibers

Obstacles preventing the use of electrospinning for mass production of drug delivery devices obviously still exist. Establishing general parameters for successful encapsulation and release of therapeutic agents is a difficult task because of the myriad of polymers, solvents, experimental conditions, and drugs which may be utilized. Basic guidelines regarding solvent properties, solution viscosities, and experimental parameters have been set forth by multiple researchers for basic and coaxial electrospinning. It is critical to determine the specific application in which the drug-loaded nanofibers will be utilized. In the instances where the therapeutic agent is not affected by the solvent used to prepare a polymer for electrospinning, basic electrospinning may be sufficient to produce the desired result. Utilization of coaxial or emulsion electrospinning to generate core-sheath-structured nanofibers may be required to construct a suitable drug delivery device if the drug or growth factor is not suitable for basic electrospinning.

The examples provided in the preceding pages represent but a small fraction of the research being conducted in the field of drug delivery using coaxial and emulsion electrospinning. In addition, multiple drug delivery technologies were combined in a number of studies to various rates of success. Sequestration of drug molecules within a carrier before production of nanofiber structures can increase the efficacy of delivery and control release profiles to great effect.^112–115^ This result may complicate matters further with respect to selection of appropriate solvents and electrospinning conditions. Particle size and composition may be critical to preservation of particle structure and release characteristics. In addition, the presence of particles within the fibers can affect the physical properties of the electrospun mats.^116–118^ Modifications to the coaxial electrospinning approaches facilitated creation of multiaxial designs and trilayer nanofibers that exhibit three distinct layers in individual fibers. Drugs may be loaded into any or all of the layers to produce systems with highly specific applications.^119–121^

## Other Aspects of Drug-Loaded Electrospun Nanofibers

All of the examples of drug-loaded nanofibers presented until now in this review rely on solvent-based methodologies to suspend therapeutic agents within solutions that are then transformed into nanofibers by the electrospinning process. Melt electrospinning is another potential technique that could be employed to include drug formulations within nanofibers. In fact, Nagy et al. demonstrated such a process when this research group loaded carvedilol into nanofibers composed of EUDRAGIT^®^ E.^122^ A solvent-based methodology was compared with melt-based electrospinning, and one finding was that the structure of carvedilol was retained in its amorphous form using both methods. The primary advantage of employing the melt-based technique developed from the lack of a solvent, thus simplifying the process and saving the cost and difficulty of dealing with various solvents. Also, the high surface area of the nanofibers permitted more rapid release of carvedilol into solution when compared with the bulk, crystalline form of the drug. An extensive review of melt electrospinning was published recently by Brown et al., exploring many general and specific applications of melt electrospinning techniques.^123^

To date, there have not been many documented attempts to include therapeutics within nanofibers fabricated using melt electrospinning. The process offers a unique concept and approach for accomplishing such an advance as it removes the requirement of a solvent, but it introduces new challenges as well. Elevated temperatures necessary for the melt electrospinning process could result, for example, in complete denaturation or deactivation of certain proteins and growth factors. For compounds that retain their activity after exposure to high temperature, melt electrospinning could be a viable option for developing nanofiber-based drug-delivery systems. Additionally, the literature is limited with respect to reports that describe the incorporation of solution-based, coaxial electrospinning methodology into melt-based electrospinning technology. McCann et al. detailed production of phase-change nanofibers consisting of a composite sheath and long-chain hydrocarbon cores that demonstrated the capability of absorbing significant quantities of thermal energy.^124^ Li et al. generated coaxial nanofibers using a melt-based system as well.^125^ The core of the nanofibers contained thermochromic compounds, which resulted in a color change to the nanofibers in response to increases or decreases in temperature. These reports could offer insight into developing coaxial, melt electrospinning systems that include growth factors and other therapeutic agents within core–shell nanofibers.

Examples have been noted in the previous paragraphs that only touch on the use of drug/growth factor-loaded nanofibers as simple, flat sheets of material. Flat materials provide some data on cellular interaction with nanofibrous sheets, but they lack the capability of producing complex, three-dimensional structures. Attempts to produce electrospun materials with greater three-dimensional complexities have met with some success in recent years. Controlled deposition of nanofibers affords the possibility of generating patterned structures in two dimensions, which could be layered to form more complex three-dimensional structures.^123^ However, the simplest examples of these experiments are seen in a number of reports detailing methods of forming small tubes composed of electrospun nanofibers with the intent of utilizing them to tissue engineer blood vessels.^19,126–129^ In the 1970s, Annis et al. detailed the creation of vascular prostheses of electrospun polyurethane nanofibers that were implanted into minipigs for up to a year.^19^ In addition to significant tissue regeneration, they noted that the mechanical properties of the synthetic vascular graft prevented formation of aneurysms. Recent attempts to create synthetic blood vessels highlight multilayering techniques as well as incorporation of natural polymers into the nanofibrous structure.^126–128^

### Three-dimensional nanofibrous scaffolds

Expansion of nanofibers to more complex three-dimensional structures is problematic. In addition to blood vessels, Gaudio et al. demonstrated fabrication of a trileaflet heart valve composed of PCL nanofibers.^129^ While the overall structure of the valve was three-dimensional, it was still composed of a two-dimensional sheet of fibers. Shim et al. showed it is possible to modify sheets of nanofibers post-production to expand them into three dimensions.^130^ Specifically, this group used a small metal comb to pull the nanofibers apart to decrease the overall nanofiber density and increase porosity of the PLLA scaffolds. Blakeney et al. approached the dimensionality issue by modifying the nanofiber collector, altering the structure of a PCL nanofiber scaffold as the nanofibers were deposited.^131^ The scaffolds in this instance were structured similar to a cotton ball and were called Focused, Low density, and Uncompressed nanoFibrous (FLUF) mesh. Jha et al. produced three-dimensional, aligned nanofiber scaffolds for nerve regeneration using a two-pole air gap technique, another modification to the nanofiber collection method.^132^ Cai et al. manipulated the conductivity of the polymer solution to create three-dimensional nanofiber structures.^133^ Hybrid scaffolds were also produced, incorporating electrospun nanofibers as a thin coating over the surfaces of pre-existing materials.^134^

An interesting extension of these approaches would incorporate drug-loaded nanofibers into more intricate, three-dimensional structures. Such systems would increase the complexity and potentially complicate determination of therapeutic agent loading and release in some instances, but the benefits may outweigh these issues for certain cases.

## Concluding Remarks

Ultimately, the intended application and its requisite components drive the development of electrospinning methods to enclose, protect, and/or control release rates for a particular drug delivery system or other purpose. This review has noted many such approaches. Over a century has passed since Boys wrote, “Fibres spun by the electrical method are so brittle that they do not seem to be of any practical use.”^1^ However, that statement was a bit premature as new applications for electrospun nanofibers are being discovered constantly in many research institutions around the globe.

## Abbreviations Used

bFGF: basic fibroblast growth factor
HA: hyaluronic acid
HFIP: hexafluoroisopropanol
NGC: nerve guidance conduit
NGF: nerve growth factor
PCL: polycaprolactone
PDLLA: poly-d,l-lactic acid
PELA: polyethylene glycol/poly-dl-lactide
PEO: polyethylene oxide
PGA: polyglycolic acid
PGS: poly(glycerol sebacate)
PLA: polylactic acid
PLGA: poly(lactic-co-glycolic acid)
PLLA: poly-l-lactic acid
SF: silk fibroin
VEGF: vascular endothelial growth factor
